# Quantified Deltoid Muscle Stiffness Can Predict Improved Muscle Strength for Elevation Following Reverse Shoulder Arthroplasty

**DOI:** 10.3390/jcm13206038

**Published:** 2024-10-10

**Authors:** Taku Hatta, Ryosuke Mashiko

**Affiliations:** Department of Orthopedic Surgery, Joint Surgery, Sports Clinic Ishinomaki, Ishinomaki 986-0850, Japan; r-mashiko@jss-clinic.com

**Keywords:** shear wave elastography, deltoid muscle stiffness, reverse shoulder arthroplasty, muscle strength, shoulder elevation

## Abstract

**Objective**: Although the indications for reverse shoulder arthroplasty (RSA) are expanding, an improvement in muscle strength in each patient following RSA remains unclear. The objective was to investigate whether or not improvement in muscle strength for shoulder elevation in patients who underwent RSA was influenced by pre- or postoperative deltoid muscle stiffness measured using shear wave elastography (SWE). **Methods**: Sixty-five patients who underwent RSA over a 12-month follow-up period were included. Patient characteristics and clinical and radiologic measurements were recorded. Preoperatively and at 3, 6, 9, and 12 months after surgery, deltoid muscle stiffness and muscle strength for scapular-plane abduction were sequentially measured using SWE and a portable dynamometer. In each quarterly period (3–6, 6–9, and 9–12 months), patients were assessed for an improvement in muscle strength and separated into two groups: improved and non-improved. To assess the risk of lack of improvement in each quarterly period, the variables were compared between the groups. **Results**: Improvement in muscle strength was observed in 52 patients (80%) at 3–6 months, 46 patients (71%) at 6–9 months, and 39 patients (60%) at 9–12 months. Notably, SWE measurements at the beginning of each period showed significantly greater values in the non-improved group than in the improved group during the subsequent quarterly period. A receiver operating characteristic (ROC) curve analysis suggested that SWE values >45.1–50.0 kPa might be associated with a lack of muscle strength improvement over 3 months with 73–87% specificity and 73–85% sensitivity. **Conclusions**: Our study demonstrated that increased deltoid muscle stiffness negatively correlated with an improvement in muscle strength following RSA. According to our results, a postoperative assessment with SWE may be useful for not only improving muscle strength after RSA but also facilitating postoperative improvement by preventing excessive stiffness in the deltoid muscle.

## 1. Introduction

Reverse shoulder arthroplasty (RSA) has been recognized as a reliable surgical option to provide satisfactory clinical outcomes for various shoulder disorders, including cuff tear arthropathy, irreparable rotator cuff tear, primary glenohumeral arthritis, rheumatoid arthritis, bone neoplasm, and comminuted proximal humerus fracture [1]. Despite the expanding indications for this procedure, an improvement in muscle strength in patients undergoing RSA remains unclear. Recent studies have investigated clinical measurements as prognostic factors for muscle strength recovery following RSA [2,3]; however, few prognostic variables for the postoperative improvement of elevated muscle strength have been identified [4].

Shear wave elastography (SWE), a novel ultrasound technique, has recently received focus to assess the mechanical properties of skeletal muscles [5,6]. A cadaveric study demonstrated that a segmental assessment using SWE could be useful for quantifying passive stiffness of the deltoid muscle [7]. Furthermore, in a recent in vivo study, SWE values measured at the anterior part of the deltoid muscle were positively correlated with postoperative pain levels in patients who underwent RSA [8].

We hypothesized that preoperative or postoperative deltoid muscle stiffness measured using SWE might be relevant to improving muscle strength for shoulder elevation following RSA. Therefore, the present study investigated whether or not the SWE values of the deltoid muscle in patients undergoing RSA could be a prognostic factor for muscle strength recovery.

## 2. Materials and Methods

### 2.1. Patient Cohort

The current study was approved by Joint Surgery, Sports Clinic Ishinomaki Institutional Review Board (approval no. JSSCI-R0603). Patients who underwent RSA at our institution and had a 12-month follow-up period were included in the study. Patients underwent surgery through the deltopectoral approach, and the arm was immobilized in an abduction brace for two weeks postoperatively. After brace removal, patients performed active and passive range of motion (ROM) exercises for forward flexion under the tutelage of therapists. Patients were instructed to perform muscle-strengthening exercises for 6–12 weeks after surgery.

For the current study, patient characteristics, including the age at surgery, sex, dominant/nondominant side, and primary shoulder disorder, routinely recorded in the clinical chart were collected. In addition, clinical assessments, including the pain level (numerical rating scale: 0–10), Constant score, and American Shoulder and Elbow Surgeon (ASES) scores, routinely examined preoperatively and at 12 months after surgery, were obtained. In addition, the active ROMs for forward flexion and external and internal rotation measured preoperatively and at 3, 6, 9, and 12 months after surgery were recorded.

### 2.2. Radiologic Assessments

Radiological factors related to RSA, the lateralization shoulder angle (LSA), and the distalization shoulder angle (DSA) were measured using an anteroposterior radiograph taken one month after surgery [9]. Two orthopedic surgeons (T.H. and R.M.) measured the LSA and DSA for each patient using the software Synapse Vincent version 6.8 (Fujifilm Inc., Tokyo, Japan), and the average value was adopted for the current analysis.

### 2.3. SWE for Deltoid Muscle Stiffness

**Figure 1:**
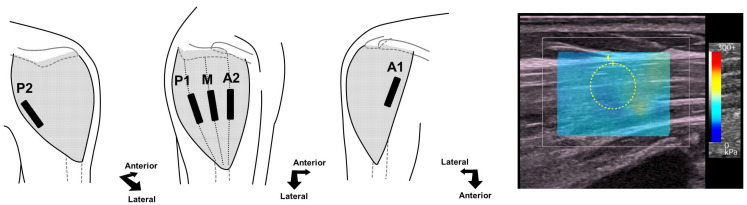
Positioning of the ultrasound probe during shear wave elastography (SWE) measurement for the deltoid muscle and an ultrasonographic image for the current assessment. SWE was examined percutaneously, and muscle stiffness values were obtained from five segments: anterior (A1, A2), middle (M), and posterior (P1, P2). The yellow dotted circle represents the region of interest for measuring muscle stiffness.

In the preoperative period and at 3, 6, 9, and 12 months postoperatively, deltoid muscle stiffness was sequentially measured using SWE (LOGIQ P10 with a linear probe, ML6-15-RS; GE Healthcare Inc., Chicago, IL, USA). We routinely performed SWE measurements for all patients who underwent RSA. The patients sat on a chair with both shoulders in a resting position. According to the previous study, to validate SWE for deltoid muscle stiffness [7], SWE measurements can be obtained from five regions (two anterior, one middle, and two posterior) of the deltoid muscle (Figure 1). Briefly, after identifying proximal and distal attachments of the deltoid muscle sonographically, the midpoint level of the muscle belly was determined for each region. The ultrasound probe was positioned on the plane parallel to the muscle fiber orientation. Regions of interest (ROI), represented by a circular area with a diameter of 5 mm, tangent to the muscle surface, were used to obtain quantitative SWE moduli. To minimize technical variation due to probe positioning or probe pressure for each region, SWE measurements were performed five times, and the average value was calculated to determine the quantified muscle stiffness. Among the values obtained from the five regions, the maximum value was used for the current analysis. For the current series, SWE values were measured by either of the two orthopedic surgeons (TH or RM).

Prior to the current analysis, we accomplished a reliability assessment for the current SWE procedure. Twenty shoulders from 10 healthy subjects (5 males and 5 females) with a mean age of 32 (range, 27–47) years old participated in the analysis. Three examiners performed SWE at an interval of one hour each. Following the steps described above, the maximal SWE values of the five regions of the deltoid muscle were obtained. Intra- and inter-examiner reliabilities were examined using intraclass correlation coefficients (ICC; ICC(1,1) and ICC(2,1), respectively). The intra- and inter-examiner reliabilities were 0.95 and 0.88 (95% confidence interval: 0.84–0.99 and 0.73–0.96), respectively. Thus, the current SWE procedure for the deltoid muscle was considered to possess satisfactory intra- and inter-examiner reliability for assessing muscle stiffness.

### 2.4. Improvement of Muscle Strength for Shoulder Elevation

Isometric muscle strength for elevation (MSE) was measured preoperatively and at 3, 6, 9, and 12 months postoperatively using a portable dynamometer (Mobie; Sakai Medical Inc., Tokyo, Japan). Patients were asked to maintain the arm position at the scapular plane with 90° or maximal angles in the shoulders with limited active elevation and to load maximal force against suppression with the dynamometer placed on the wrist. During the postoperative quarterly periods of 3–6, 6–9, and 9–12 months, patients were assessed for the presence or absence of improvement in MSE. For each quarterly period, patients were separated into two groups: “improved” and “non-improved”. To investigate the variables correlating with the improvement during each quarterly period, the patient’s characteristics, implant types, radiologic measurements, and SWE values at the preoperative and postoperative examinations were compared between the improved and non-improved groups.

### 2.5. Statistical Analyses

Statistical analyses were performed using an unpaired *t*-test, Pearson’s chi-square test, or Fisher’s exact test to compare variables between groups. Intra- and inter-observer reliability was examined using an intraclass correlation coefficient (ICC; ICC(1,1) and ICC(2,1), respectively). In addition, analyses of accuracy, sensitivity, specificity, the receiver operating characteristic (ROC), and area under the ROC curve (AUC) were performed to calculate cut-off values. Optimal diagnostic cut-off values were determined using the Youden Index from the ROC curve. The unpaired *t*-test, Pearson’s chi-square test, Fisher’s exact test, and the analysis for the ROC curve were performed with GraphPad Prism 5.0 (GraphPad Software, La Jolla, CA, USA). The analyses for intra- and inter-examiner reliability were performed with SPSS 18.0 (IBM, Armonk, NY, USA). Statistical significance was set at *p* < 0.05.

## 3. Results

Among the 65 patients, an improvement in MSE was observed in 52 patients (80%) at 3–6 months, 46 patients (71%) at 6–9 months, and 39 patients (60%) at 9–12 months. For each quarterly period, the patient characteristics showed no correlation with the improvement in MSE (Table 1). Regarding preoperative clinical variables, active ROM for forward flexion positively correlated with an improvement in MSE at 3–6 months (*p* = 0.02), 6–9 months (*p* = 0.04), and 9–12 months (*p* = 0.004, Table 2).

In addition, preoperative MSE and Constant scores were significantly associated with MSE improvements at 9–12 months (*p* = 0.007 and 0.02, respectively). In contrast, no postoperative clinical variables or radiologic measurements (LSA or DSA) were correlated with MSE improvements during each quarterly period (Table 3 and Table 4).

**Figure 2:**
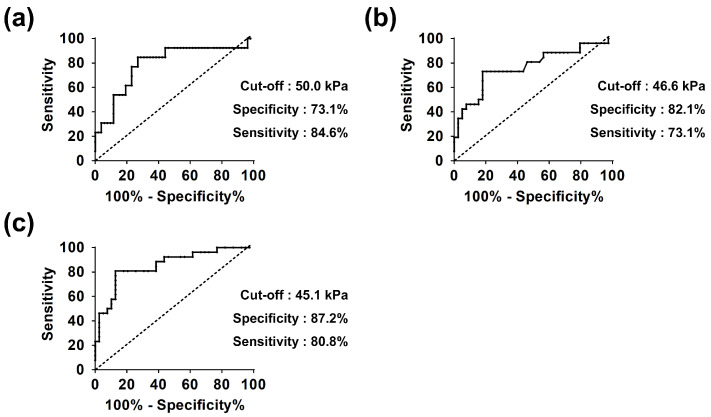
The receiver operating characteristic (ROC) curve for the cut-off SWE values leading to a lack of improvement in muscle strength for elevation (MSE). The figures represent the SWE measurements at the beginning of the postoperative period when an MSE improvement was expected during the subsequent 3-month period: at 3 months for 3–6 months (**a**), at 6 months for 6–9 months (**b**) and at 9 months for 9–12 months (**c**).

Although preoperative SWE showed no correlation with MSE improvement, postoperative SWE values measured at 3, 6, 9, and 12 months were partially associated with MSE improvement during quarterly periods. In particular, SWE values measured at the beginning of each quarterly period were significantly greater in the non-improved group than in the improved group (3-month SWE mean 67.0 vs. 44.9 kPa; *p* < 0.001 for 3–6 months period, 6-month SWE 51.2 vs. 38.3 kPa; *p* < 0.001 for 6–9 months period, 9-month SWE 54.6 vs. 37.3 kPa; and *p* < 0.001 for 9–12 months period, Table 4). The AUC of 3-month SWE for 3–6 months period, 6-month SWE for 6–9 months, and 9-month SWE for 9–12 months were 0.79, 0.77, and 0.85, respectively. The ROC curve analysis showed that patients with SWE values greater than 50.0, 46.6, and 45.1 kPa at 3, 6, and 9 months experienced improvements in MSE during the subsequent 3-month period with 75.3%, 78.5%, and 84.6% accuracy, 73.1%, 82.1%, and 87.2% specificity and 84.6%, 73.1%, and 80.8% sensitivity, respectively (Figure 2).

## 4. Discussion

The current study investigated the effect of preoperative and postoperative deltoid muscle stiffness measured with SWE on the improvement of muscle strength for shoulder elevation in patients undergoing RSA, with the aim of identifying prognostic factors for gaining muscle strength. Notably, our results demonstrate that quantified deltoid muscle stiffness measured at postoperative periods of 3, 6, and 9 months significantly correlates with the improvement in muscle strength assessed 3 months later. By contrast, no radiologic variables to indicate distalization and lateralization were relevant to postoperative gains in muscle strength. Accordingly, excessive deltoid muscle stiffness may be an independent risk factor that impedes the muscle strength recovery of shoulder elevation during the subsequent three-month follow-up period. To our knowledge, this is the first study to demonstrate the importance of mechanical properties of the deltoid muscle for postoperative functional recovery in patients undergoing RSA.

It is well known that biomechanical alterations are dramatically present in shoulders with RSA, including an increased moment arm between the deltoid muscle and stabilized center of rotation medially and inferiorly onto the scapula [10]. Although a large number of clinical reports have shown satisfactory improvement in patients undergoing RSA [2,11,12], alterations in periscapular muscle activities during shoulder motion have not yet been clarified. In vitro and in silico studies have advocated biomechanically beneficial alterations following RSA in terms of facilitating deltoid muscle activities [13,14,15]; however, a recent in vivo analysis to quantify periscapular muscle activities using positron emission tomography demonstrated that significantly increased muscle activities of the anterior, middle, and posterior parts of the deltoid muscle were required for shoulder elevation in patients who underwent RSA compared to those with an intact shoulder [16]. Accordingly, repetitively loaded forces in the deltoid muscle with RSA may provide excessive stress in the muscle belly or acromion/scapular spine attached to the muscle, resulting in pathological changes, such as fatigue-related dysfunction in the deltoid muscle or stress fractures in the scapula during mid- to long-term periods. To identify the extent of this damage in each patient undergoing RSA, we conducted the current study based on the hypothesis that SWE might be helpful for quantifying mechanical properties and identifying pathological alterations of the deltoid muscle in each patient undergoing RSA.

SWE has recently received attention for assessing stiffness in skeletal muscles, with increased application in clinical practice, such as an assessment tool for the severity of muscular injuries or postoperative functional recovery. Pimenta et al. [17] investigated the effect of repeated sprint tasks on SWE changes in skeletal muscles and demonstrated a significant change after the sprint task in the biceps femoris muscle with a mean increase of 10%. Kumamoto et al. [18] investigated SWE changes for the lumbodorsal muscle fatigue task and demonstrated a post-task increase in mean SWE values measured at the multifidus muscle. Therefore, in our study, the increased SWE values observed in the non-improved group might represent altered conditions associated with repetitive stress and fatigue in the deltoid muscle in patients undergoing RSA.

Postoperative muscle strength has recently received focus in the context of functional recovery in patients undergoing RSA. Alta et al. [19] investigated torque strength in patients who underwent RSA with a mean follow-up of 23 months and indicated a relatively decreased muscle strength in abduction and adduction, with 19–78% of normal subjects. They suggested that limited muscle strength might lead to reduced postoperative ROMs. Wang et al. [20] investigated the torque strength with regard to the level of participation in sports and recreation in patients undergoing RSA with a minimal follow-up of 12 months and found that postoperative torque strength positively correlated with the level of sports participation and the patient-reported quality of daily living activities. Müller et al. [2] focused on the glenosphere size in patients with RSA and showed a positive correlation between the diameter and postoperative muscle strength in abduction at one-year follow-up. Almeida et al. [4] investigated patients’ characteristics, radiologic severity, and clinical scores to clarify the prognostic factors in gaining deltoid muscle strength in patients undergoing RSA, finding that no preoperative variables were prognostic factors. Since no assessment index has been established for recovery in deltoid muscle strength, surgeons should carefully perform follow-up with a focus on the progress of muscle strength in each patient.

Several limitations associated with the present study warrant mention. First, we assessed isometric muscle strength in shoulders in a single position (scapular-plane abduction at 90°) in this study. The current position has been applied in several biomechanical studies to investigate the elevation of muscle strength [16]. In addition, a recent study investigated shoulder elevation angles during daily activities in patients undergoing RSA using inertial measurement units and demonstrated that more than 99% of the day could be spent with up to 90° of shoulder elevation [21]. However, a further assessment of various shoulder motions is important to confirm the usefulness of SWE for the assessment of postoperative muscle function. Second, the follow-up period was relatively short (one year). Although the current study investigated postoperative improvement in patients undergoing RSA, further studies with longer follow-up periods are warranted. Third, we adopted the maximal values of SWE measurements to assess the correlation to MSE improvement following RSA. Although previous studies have indicated an increase in skeletal muscle stiffness measured with SWE as repetitive tasks or chronic muscle fatigues [17,18], it remains unclear whether or not decreased muscle stiffness or heterogeneity within the muscle could have any potential impacts on the alteration of muscle strength. Fourth, our cohort consisted of patients who underwent surgery in a single institution, limiting the generalizability of our results, such as surgical techniques, physical therapy protocol, or the percentage of patients whose muscle strength improved postoperatively.

The strength of the current study was to reveal the relationship between postoperative MSE improvement and the assessment of deltoid muscle stiffness using SWE, which could indicate potential clinical applications for patients with RSA. In particular, the current study, which comprised a longitudinal assessment during a follow-up of one year, could elucidate the clinical applicability of the muscle stiffness measurement for RSA and the importance of deltoid muscle stiffness in postoperative MSE improvement. Based on these results, future studies would be essential to investigate the efficacy of postoperative management that could reduce excessive deltoid muscle stiffness in patients with high muscle stiffness values for postoperative muscle strength recovery.

## 5. Conclusions

In conclusion, we demonstrated that increased deltoid muscle stiffness negatively correlated with subsequent improvements in muscle strength during the postoperative period. According to our results, a postoperative assessment with SWE may be useful for not only improving muscle strength after RSA but also facilitating postoperative improvement by preventing excessive stiffness in the deltoid muscle.

## Figures and Tables

**Table 1:** Patient characteristics for improvement in MSE during each quarterly period.

	3–6 Months			6–9 Months			9–12 Months		
	Improved	Non-Improved	*p* Value	Improved	Non-Improved	*p* Value	Improved	Non-Improved	*p* Value
Age, mean (SD), year	75 (6)	75 (9)	0.65	76 (6)	73 (4)	0.07	74 (5)	76 (6)	0.07
Sex, female/male	35/17	9/4	1.00	33/13	11/8	0.38	25/14	19/7	0.59
Dominant/non-dominant side	40/12	8/5	0.30	36/10	12/7	0.23	29/10	19/7	1.00
Primary shoulder disorders									
Cuff tear arthropathy	44	11	0.63	38	17	0.46	34	21	0.61
Osteoarthritis	6	2		7	1		4	4	
Rheumatoid arthritis	2			1	1		1	1	
Implant type									
Comprehensive-TM Reverse	42	7	0.08	36	13	0.26	31	18	0.37
Arrow	7	3		5	5		6	4	
Medacta Shoulder System	3	3		5	1		2	4	

MSE, muscle strength for elevation; TM, trabecular metal; and SD, standard deviation.

**Table 2:** Preoperative clinical variables for improvement in MSE during each quarterly period.

	3–6 Months			6–9 Months			9–12 Months		
	Improved	Non-Improved	*p* Value	Improved	Non-Improved	*p* Value	Improved	Non-Improved	*p* Value
Active ROM									
Forward flexion (degree)	101 (34)	75 (37)	0.02	101 (36)	83 (31)	0.04	106 (34)	81 (35)	0.004
External rotation (degree)	15 (18)	8 (19)	0.24	15 (18)	11 (18)	0.57	16 (15)	10 (23)	0.18
Internal rotation (score)	3.4 (1.8)	3.2 (2.2)	0.78	3.5 (2.0)	3.1 (1.8)	0.30	3.6 (1.9)	3.0 (2.0)	0.18
Pain (NRS, 0–10)	6.1 (2.2)	6.5 (2.6)	0.52	6.1 (2.4)	6.4 (1.8)	0.54	6.1 (2.2)	6.3 (2.4)	0.69
Muscle strength for elevation (kgf)	2.1 (1.5)	2.3 (1.5)	0.62	2.4 (1.5)	2.1 (1.1)	0.28	2.7 (1.7)	1.7 (0.9)	0.007
Constant score	41.3 (12.0)	36.4 (11.1)	0.18	41.7 (11.8)	36.9 (9.2)	0.20	43.1 (11.9)	36.2 (10.8)	0.02
ASES score	43.1 (19.4)	36.9 (18.0)	0.57	43.7 (18.7)	41.0 (17.3)	0.34	43.3 (19.0)	41.1 (19.4)	0.65

MSE, muscle strength for elevation; ROM, range of motion; NRS, numerical rating scale; and ASES, American Shoulder and Elbow Surgeons.

**Table 3:** Postoperative clinical variables for improvement in MSE during each quarterly period.

	3–6 Months			6–9 Months			9–12 Months		
	Improved	Non-Improved	*p* Value	Improved	Non-Improved	*p* Value	Improved	Non-Improved	*p* Value
Postop. 3 months									
Active ROM									
Forward flexion (degree)	109 (22)	107 (27)	0.80	110 (25)	106 (18)	0.49	110 (24)	106 (22)	0.49
External rotation (degree)	19 (13)	20 (24)	0.70	19 (17)	18 (13)	0.76	19 (14)	19 (19)	0.94
Internal rotation (score)	2.7 (1.5)	3.4 (1.8)	0.17	2.8 (1.5)	3.1 (1.7)	0.53	3.0 (1.4)	2.7 (1.5)	0.38
Muscle strength for elevation (kgf)	3.4 (1.9)	3.3 (1.3)	0.56	3.5 (1.5)	3.1 (1.3)	0.17	3.4 (1.5)	2.9 (1.2)	0.13
Postop. 6 months									
Active ROM									
Forward flexion (degree)	123 (19)	117 (19)	0.33	123 (20)	119 (16)	0.34	123 (18)	118 (21)	0.31
External rotation (degree)	24 (16)	27 (16)	0.62	25 (18)	25 (10)	0.98	26 (13)	24 (20)	0.54
Internal rotation (score)	3.7 (2.0)	3.7 (1.3)	1.00	3.5 (1.8)	4.1 (1.9)	0.31	3.8 (1.6)	3.3 (2.0)	0.32
Muscle strength for elevation (kgf)	4.0 (1.6)	3.2 (1.8)	0.12	3.8 (1.5)	4.1 (1.8)	0.22	4.1 (1.8)	3.4 (1.3)	0.11
Postop. 9 months									
Active ROM									
Forward flexion (degree)	126 (20)	122 (22)	0.53	126 (20)	126 (22)	1.00	127 (21)	122 (21)	0.36
External rotation (degree)	28 (16)	29 (16)	0.79	27 (17)	29 (13)	0.65	32 (14)	23 (18)	0.02
Internal rotation (score)	4.2 (2.1)	4.2 (1.4)	0.97	4.1 (2.1)	4.4 (2.1)	0.59	4.7 (1.7)	3.4 (2.0)	0.005
Muscle strength for elevation (kgf)	4.1 (1.6)	3.9 (1.7)	0.69	4.1 (1.6)	3.9 (1.7)	0.62	4.1 (1.8)	3.8 (1.4)	0.45
Postop. 12 months									
Active ROM									
Forward flexion (degree)	129 (18)	123 (18)	0.32	129 (19)	124 (17)	0.65	131 (17)	123 (19)	0.10
External rotation (degree)	29 (16)	29 (15)	0.93	29 (17)	29 (13)	0.85	34 (13)	22 (18)	0.003
Internal rotation (score)	4.4 (2.2)	4.2 (1.5)	0.72	4.5 (2.0)	4.1 (2.3)	0.31	4.9 (1.9)	3.5 (2.1)	0.01
Pain (NRS, 0–10)	1.4 (2.0)	1.8 (1.7)	0.24	1.5 (2.0)	1.5 (1.9)	0.96	1.2 (1.5)	1.9 (2.4)	0.18
Muscle strength for elevation (kgf)	4.2 (1.6)	3.6 (1.6)	0.46	4.2 (1.6)	3.8 (1.9)	0.35	4.6 (1.6)	3.3 (1.3)	<0.001
Constant score	66.1 (10.4)	62.2 (8.9)	0.22	67.1 (8.5)	64.6 (10.8)	0.37	68.2 (8.7)	61.0 (10.9)	0.005
ASES score	83.8 (17.1)	81.1 (13.5)	0.60	84.8 (16.6)	81.6 (16.4)	0.62	86.4 (13.5)	78.5 (19.3)	0.06

MSE, muscle strength for elevation; ROM, range of motion; NRS, numerical rating scale; and ASES, American Shoulder and Elbow Surgeons.

**Table 4:** Radiologic and SWE measurements for improvement in MSE during each quarterly period.

	3–6 Months			6–9 Months			9–12 Months		
	Improved	Non-Improved	*p* Value	Improved	Non-Improved	*p* Value	Improved	Non-Improved	*p* Value
Plain radiograph									
Postop. DSA	45.6 (6.8)	47.5 (8.0)	0.37	45.5 (7.6)	46.7 (6.3)	0.52	45.5 (6.2)	46.6 (8.2)	0.56
Postop. LSA	86.9 (6.3)	87.9 (7.4)	0.64	87.3 (7.2)	86.7 (5.3)	0.72	88.2 (5.8)	85.5 (7.1)	0.10
SWE									
Preop.	42.4 (20.4)	50.5 (17.9)	0.20	42.1 (20.0)	51.2 (17.7)	0.07	40.5 (16.4)	49.4 (23.9)	0.08
Postop. 3 months	44.9 (16.8)	67.0 (23.0)	<0.001	43.0 (13.4)	58.8 (24.6)	0.001	46.1 (18.7)	54.2 (21.4)	0.11
Postop. 6 months	42.5 (15.5)	47.2 (13.4)	0.32	38.3 (10.6)	51.2 (17.7)	<0.001	43.7 (17.5)	43.0 (11.2)	0.86
Postop. 9 months	41.1 (15.0)	56.7 (22.0)	0.004	42.2 (17.3)	47.3 (17.9)	0.25	37.3 (11.9)	54.6 (19.7)	<0.001
Postop. 12 months	36.2 (11.7)	45.8 (16.7)	0.02	36.0 (11.7)	41.3 (15.0)	0.12	33.9 (11.6)	44.4 (13.3)	0.001

SWE, shear wave elastography; MSE, muscle strength for elevation; DSA, dorsalization shoulder angle; and LSA, lateralization shoulder angle.

## Data Availability

Data are contained within the article.
